# Emergency Diagnostic Phenotypes and Differential Survival in Colorectal Cancer: A Real-World Cohort Study

**DOI:** 10.3390/diagnostics16162487

**Published:** 2026-08-07

**Authors:** Alexandru-Marian Vieru, Sergiu-Marian Cazacu, Maria-Lorena Mustață, Virginia-Maria Rădulescu, Petrică Popa, Tudorel Ciurea

**Affiliations:** 1 Doctoral School, University of Medicine and Pharmacy of Craiova, 200349 Craiova, Romania; alexandruvieru1993@gmail.com (A.-M.V.); umlorena@yahoo.com (M.-L.M.); 2 Faculty of Medicine, University of Medicine and Pharmacy of Craiova, 200349 Craiova, Romania; virginia.radulescu@umfcv.ro (V.-M.R.); popa.petrica@umfcv.ro (P.P.); tudorel.ciurea@umfcv.ro (T.C.)

**Keywords:** colorectal cancer, emergency presentation, bowel obstruction, perforation, lower gastrointestinal bleeding, risk stratification, overall survival, treatment allocation, real-world evidence

## Abstract

**Background/Objectives**: Emergency presentation in colorectal cancer (CRC) is commonly regarded as a uniformly high-risk condition, although clinically distinct emergency phenotypes may carry substantially different prognostic implications. We aimed to characterise emergency diagnostic phenotypes, bowel obstruction, perforation, severe stenosis, and lower gastrointestinal bleeding, and to examine their associations with treatment allocation, perioperative mortality, and overall survival after adjustment for recorded covariates. **Methods**: We performed a retrospective, real-world cohort study of 1051 consecutive patients with CRC diagnosed between 2018 and 2021 at a tertiary referral centre. Patients were assigned to four mutually exclusive groups using a classification defined before outcome analysis (obstructive–perforative, stenotic, haemorrhagic, elective). Overall survival was assessed using Kaplan–Meier analysis and multivariable Cox regression, with formal testing of the proportional hazards assumption. **Results**: Emergency presentation occurred in 43.7% of patients but was markedly heterogeneous. Metastatic burden did not differ across phenotypes (*p* = 0.122). The obstructive–perforative phenotype showed the least favourable outcomes: perioperative mortality was 11.5%, and it remained associated with mortality after adjustment for recorded covariates (HR 1.58, 95% CI 1.25–2.00; *p* < 0.001), with hazard ratios ranging from 1.33 to 1.69 across six model specifications. Haemorrhagic presentation was rectal-predominant and behaved favourably, with survival comparable to elective diagnosis. Among 30-day survivors, the excess hazard was attenuated but persisted (HR 1.39, 95% CI 1.04–1.86). **Conclusions**: Emergency presentation in CRC is not a monolithic risk category. Recognising the presenting phenotype may offer a pragmatic framework for provisional risk characterisation at diagnosis, but external validation and adjustment for patient-level comorbidity and performance status are required before routine clinical use.

## 1. Introduction

Colorectal cancer (CRC) remains one of the most common malignancies and a leading cause of cancer-related death worldwide, accounting for close to two million new cases and approaching one million deaths each year [1]. Its prognosis is strongly stage-dependent: patients identified with localised disease are frequently curable, whereas those reaching medical attention with advanced or disseminated disease face markedly poorer outcomes. In the absence of, or with incomplete uptake of, organised screening, a substantial proportion of patients continue to be diagnosed only after symptoms appear, and a considerable minority present acutely, as a surgical emergency.

Emergency presentation has long been recognised as a marker of adverse outcome in colorectal cancer. Roughly one in five patients presents in this way, most commonly with bowel obstruction or perforation [2], and such presentations are consistently associated with more advanced stages, higher operative mortality, and poorer long-term survival than elective, investigation-led diagnosis [3,4]. Obstruction and perforation are, moreover, regarded as adverse prognostic features in their own right, independent of tumour stage, and figure among the high-risk criteria that inform adjuvant decision-making [5,6]. Large multicentre audits of malignant large-bowel obstruction have similarly documented substantial perioperative mortality and identified physiological and operative risk factors that shape early outcomes [7].

Despite this, “emergency presentation” is most often handled analytically as a single, monolithic category—emergency vs. elective—an approach that risks obscuring clinically distinct entities. The acute presentations of colorectal cancer are mechanistically heterogeneous. Obstruction and perforation represent an acute mechanical, and often septic, catastrophe that mandates urgent operation in an unprepared field; lower gastrointestinal bleeding, by contrast, is frequently an early alarm symptom that brings patients to attention sooner and is associated with shorter diagnostic intervals and, often, more distal, earlier-stage disease [8]; and severe stenosis occupies an intermediate, sub-occlusive position. Direct comparisons within the acute spectrum remain relatively few and have largely been confined to perforation vs. obstruction, with broadly similar overall survival but greater morbidity attributed to perforation [9,10]. Few studies have modelled the full range of emergency phenotypes within a single consecutive cohort, or tested whether they carry differential, stage-independent prognostic weight—precisely the questions most relevant to risk stratification at the point of presentation.

Two further considerations bear on such an analysis. First, primary tumour location is now an established prognostic and predictive axis in colorectal cancer, with left-sided tumours conferring a modest but robust survival advantage over right-sided tumours [11,12,13]. Because the mode of presentation and tumour site are related, any assessment of phenotype must account for location. Second, our study window (2018–2021) spans the onset of the COVID-19 pandemic, which disrupted colorectal diagnostic and referral pathways, reduced screening and endoscopy, and was associated with a shift towards more acute and more advanced presentations [14,15]. The diagnostic period, therefore, represents a relevant covariate rather than an exposure of interest.

Against this background, we conducted a real-world cohort study of consecutive patients with colorectal cancer managed at a Romanian tertiary referral centre. Our aims were threefold: (i) to characterise the distribution of emergency diagnostic phenotypes—bowel obstruction, perforation, severe stenosis, and lower gastrointestinal bleeding—at diagnosis; (ii) to compare their clinicopathological profile, treatment allocation, and perioperative mortality; and (iii) to determine whether these phenotypes are independently associated with overall survival, using a mutually exclusive classification defined before outcome analysis and adjusting for stage, tumour location, and diagnostic period. We hypothesised that emergency presentation is not a uniform, high-risk label but a heterogeneous set of phenotypes with divergent—and clinically actionable—prognostic implications.

## 2. Materials and Methods

### 2.1. Study Design and Setting

This retrospective observational cohort study was conducted at the Oncology and Surgical Departments of the Craiova County Emergency Hospital, a tertiary referral centre for southern Romania. The study included consecutive patients newly diagnosed with colorectal cancer between January 2018 and December 2021. Data were drawn from the institutional oncology registry, which prospectively records newly diagnosed digestive malignancies, and were supplemented by review of electronic medical records and endoscopy, surgical, and pathology reports for variables not captured in structured fields. The aim was to characterise emergency diagnostic phenotypes at presentation and to evaluate their association with clinicopathological features, treatment allocation, perioperative mortality, and overall survival.

### 2.2. Study Population and Eligibility

Eligible participants were adults with a new diagnosis of histologically confirmed colorectal carcinoma/adenocarcinoma documented within the study window and managed through institutional clinical pathways. Patients were excluded if the diagnosis was not histologically confirmed, if the tumour was non-colorectal or non-epithelial, or if core information required to classify the mode of presentation, treatment allocation, or vital status was missing. After applying these criteria, 1051 patients constituted the analytic cohort. For time-to-event analyses, a subset of 862 patients with a datable survival interval was used (see Section 2.4), and multivariable survival models were fitted in 859 of these, three patients having no recorded age. Denominators are reported explicitly throughout.

This study was not conducted under a registered protocol. The phenotype definitions, the severity hierarchy, and the covariate set were nevertheless specified before any outcome data were analysed, and the robustness analyses reported in Section 3.7 are designated exploratory.

### 2.3. Emergency Diagnostic Phenotypes: Operational Definitions

An emergency diagnostic presentation was defined as the presence, at or immediately preceding diagnosis, of at least one of four clinically defined phenotypes:

• Bowel obstruction—Clinical and/or imaging evidence of complete or high-grade large-bowel obstruction attributable to the tumour;
• Perforation—Tumour-related free perforation or contained perforation/abscess;
• Severe stenosis—An endoscopically impassable or sub-occlusive stenosing tumour without frank obstruction;
• Lower gastrointestinal bleeding (LGIB)—Overt rectal bleeding or symptomatic anaemia requiring urgent evaluation or transfusion attributable to the tumour.

Because these phenotypes reflect distinct pathophysiological mechanisms and because a minority of patients presented with more than one, patients were assigned to four mutually exclusive groups using a severity hierarchy (perforation/obstruction > severe stenosis > LGIB > elective):

1. Elective—None of the four emergency phenotypes;
2. Haemorrhagic—LGIB, without obstruction, perforation, or severe stenosis;
3. Stenotic—Severe stenosis, without obstruction or perforation;
4. Obstructive–perforative—Bowel obstruction and/or perforation.

Obstruction and perforation were combined into a single “obstructive–perforative” category on both mechanistic grounds (acute mechanical/catastrophic luminal events requiring urgent operative management) and because of the small number of isolated perforations. The distribution of individual phenotypes (allowing overlap) is reported descriptively alongside the mutually exclusive grouping.

The hierarchy was a prespecified, deterministic coding rule rather than a validated physiological severity score. Obstruction and perforation were prioritised because they represent acute mechanical or septic complications that commonly require immediate intervention and are recognised as adverse clinical features; severe stenosis was placed next as a sub-occlusive state without frank obstruction; and bleeding was assigned only when none of these higher-acuity mechanical complications was present [2,5,6,7,16]. Its purpose was to prevent double counting and ensure reproducible assignment of overlapping presentations, not to assert a universal ordering of prognosis.

### 2.4. Variables and Outcome Definitions

Variables were abstracted into a structured dataset (Microsoft Excel, Microsoft Corp., Redmond, WA, USA) and comprised:

• Demographics: Age at diagnosis and sex.
• Tumour characteristics: TNM components (AJCC/UICC 8th edition), stage group, metastatic status (M0/M1; Mx where undocumented), histological grade, and lymphovascular/perineural invasion when recorded.
• Anatomical sidedness: Classified from the documented tumour location as right-sided (caecum, ascending, transverse colon), left-sided (descending, sigmoid colon), or rectal (rectum/anorectal); synchronous or unclassifiable locations were grouped separately.
• Treatment allocation: Any surgical intervention (yes/no) and treatment intent classified as radical (curative-intent resection), palliative, or nonoperative. A composite non-curative allocation endpoint combined palliative surgery and nonoperative management.
• Perioperative mortality: In-hospital or 30-day death among operated patients.
• Diagnostic period: Pre-pandemic (2018–2019) vs. pandemic (2020–2021). Metastatic status and stage group were recorded independently in the source records and were discordant in nine patients (six coded M1 without stage IV; three coded stage IV without M1); a further 54 patients had Mx, so M-based and stage-based denominators differ slightly.

Overall survival (OS) was defined from the date of diagnosis to the date of death (event) or last documented oncological contact (censored). Of the 1051 patients, 862 had a datable time-to-event and formed the survival cohort (433 deaths, 429 censored; median follow-up in survivors 1731 days). A total of 189 patients known to have died but without a recorded death date could not be placed on the time axis and were excluded from time-to-event analyses; the consequences of their exclusion are examined directly in Section 3.7. Same-day deaths (death recorded on the diagnosis date) were retained as genuine perioperative events (assigned a nominal 0.5-day survival) rather than censored at time zero, a choice appropriate to a surgical emergency cohort in which same-day mortality is a real clinical outcome; the alternative rule is tested in Section 3.7.

For these 189 patients, the retrospective analytic dataset retained the fact of death but contained no retrievable calendar date from which an event time could be calculated. No independent date source was available within the study dataset, and the specific clinical or administrative pathway leading to the absent dates could not be reconstructed retrospectively. Consequently, these patients could not be positioned validly on a Kaplan–Meier time axis.

### 2.5. Statistical Analysis

Continuous variables are reported as mean ± standard deviation (SD) or median (interquartile range, IQR), as appropriate; categorical variables as counts and percentages. Between-group comparisons across the four phenotypes used one-way ANOVA or the Kruskal–Wallis test for continuous variables and the χ^2^ test for categorical variables.

The primary endpoint was overall survival. Secondary endpoints were perioperative mortality among patients who underwent surgery and non-curative treatment allocation. Metastatic disease at diagnosis (M1) was analysed as a mechanistic endpoint to test whether phenotypic effects operate through stage. All robustness analyses described below were exploratory.

Overall survival was estimated by the Kaplan–Meier method and compared across phenotypes with the log-rank test, both globally and pairwise against the elective reference. Independent predictors of mortality were assessed using multivariable Cox proportional hazards regression (enter method). Covariates were selected a priori on clinical grounds rather than by stepwise data-driven procedures: phenotype group (reference: elective), age, sex, metastatic status, tumour sidedness, and diagnostic period. Surgery was deliberately excluded from the primary model. Because the decision to operate lies on the causal pathway between an acute presentation and death—obstruction and perforation compel urgent surgery, and that surgery carries its own perioperative mortality—conditioning on it would constitute adjustment for a mediator, a recognised source of overadjustment bias, and would attenuate the very effect under study [17]. The primary model, therefore, estimates the total effect of phenotype. A sensitivity model, which additionally includes surgery and estimates the direct effect, is reported alongside it. The proportional hazards assumption was tested formally using scaled Schoenfeld residuals [18]. Metastatic status (*p* < 0.001) and sex (*p* = 0.006) violated the assumption, and age was borderline (*p* = 0.058); the three phenotype terms did not (*p* = 0.41–0.48). Accordingly, the primary model was stratified by metastatic status, sex, and age group (<65, 65–74, ≥75 years), after which no covariate violated the assumption of linearity. A complementary unstratified model, in which all covariates are assigned hazard ratios, is also reported; for metastatic status and sex, these represent time-averaged effects. Model discrimination was quantified by Harrell’s concordance index, with optimism corrected through bootstrap resampling [19]; the concordance index of a stratified model is not directly comparable with that of an unstratified model, because the strata absorb the strongest predictors.

Two complementary multivariable logistic regression models were fitted: one for metastatic disease at diagnosis and one for non-curative allocation, each adjusted for age, sex, diagnostic period, and—where it was not the outcome—metastatic status. Effect sizes are reported as hazard ratios (HRs) or odds ratios (ORs) with 95% confidence intervals (CIs). All tests were two-sided, with α = 0.05.

A complete-case approach was applied to each analysis, without imputation, and denominators are reported for every model. Imputation was not attempted for the principal source of missingness—patients known to have died but without a recorded date of death—because the missing quantity is the event time rather than a covariate, and imputing it would fabricate the outcome the study seeks to measure. The consequences of this choice are examined directly in Section 3.7. Reporting follows the STROBE recommendations for observational studies [20]. Descriptive statistics and univariable group comparisons were performed in IBM SPSS Statistics v26 (IBM Corp., Armonk, NY, USA). Survival modelling, proportional hazards testing, logistic regression, and bootstrap internal validation were performed in Python version 3.9.13 (lifelines version 0.27.4, statsmodels version 0.13.5, and SciPy version 1.9.3 ). Data preprocessing was undertaken in Microsoft Excel (Microsoft Corp., Redmond, WA, USA).

### 2.6. Ethics

The study received approval from the Ethics Committee of the University of Medicine and Pharmacy of Craiova (approval number 18593/13 April 2023) and was conducted in accordance with the principles of the Declaration of Helsinki. Given the retrospective design, the exclusive use of routinely collected clinical data, and prior anonymisation of all identifiers before analysis, the requirement for individual written informed consent was waived by the Ethics Committee.

## 3. Results

### 3.1. Cohort and Distribution of Emergency Diagnostic Phenotypes

A total of 1051 consecutive patients with colorectal cancer diagnosed between 2018 and 2021 were analysed. The mean age was 67.3 ± 10.6 years, and 59.9% were men. An emergency diagnostic presentation—defined as at least one of bowel obstruction, perforation, severe stenosis, or lower gastrointestinal bleeding at diagnosis—was recorded in 459 patients (43.7%). Considered individually (categories not mutually exclusive), lower gastrointestinal bleeding was the most frequent phenotype (177 patients, 16.8%), followed by obstruction (156, 14.8%), stenosis (123, 11.7%), and perforation (41, 3.9%); 36 patients (7.8% of emergencies) presented with more than one phenotype.

Because these phenotypes reflect distinct pathophysiological mechanisms, patients were assigned to four mutually exclusive groups using a severity-based hierarchy (perforation/obstruction > stenosis > bleeding > elective): elective (592, 56.3%), haemorrhagic (170, 16.2%), stenotic (95, 9.0%), and obstructive–perforative (194, 18.5%). This structure was used for all subsequent comparative analyses.

### 3.2. Clinicopathological Profile Across Phenotypes

Baseline demography was comparable across the four groups, with no significant differences in age (*p* = 0.364) or sex (*p* = 0.639). Notably, the burden of metastatic disease at diagnosis was also statistically similar across phenotypes: the proportion of M1 disease ranged from 21.2% (haemorrhagic) to 33.7% (stenotic) and did not differ significantly (*p* = 0.122), and the same was true for stage IV disease (*p* = 0.072). In other words, emergency presentation as a whole did not track a higher metastatic burden.

The strongest phenotype-related difference was anatomical (*p* < 0.001). Haemorrhagic presentations were predominantly rectal (51.8%), whereas obstructive–perforative cancers were predominantly left-sided colonic (52.6%) with comparatively few rectal tumours (21.1%). High-grade histology (G3–G4) was somewhat more frequent in the obstructive–perforative group (27.3%) than in the haemorrhagic group (18.2%). Baseline and clinicopathological characteristics are summarised in Table 1.

### 3.3. Treatment Allocation and Perioperative Mortality

Treatment allocation varied significantly across phenotypes (surgery rate, *p* < 0.001). Surgical resection was near-universal in the obstructive–perforative group (93.8%), reflecting the need for urgent operative management, and lower in the haemorrhagic (65.9%), elective (70.6%), and stenotic (78.9%) groups. Conversely, non-curative allocation (palliative surgery or no surgery) was most frequent in the haemorrhagic group (41.8%), a pattern driven by the predominance of rectal tumours managed along nonoperative or neoadjuvant pathways rather than by a higher metastatic burden (see Section 3.6).

**Figure 1:**
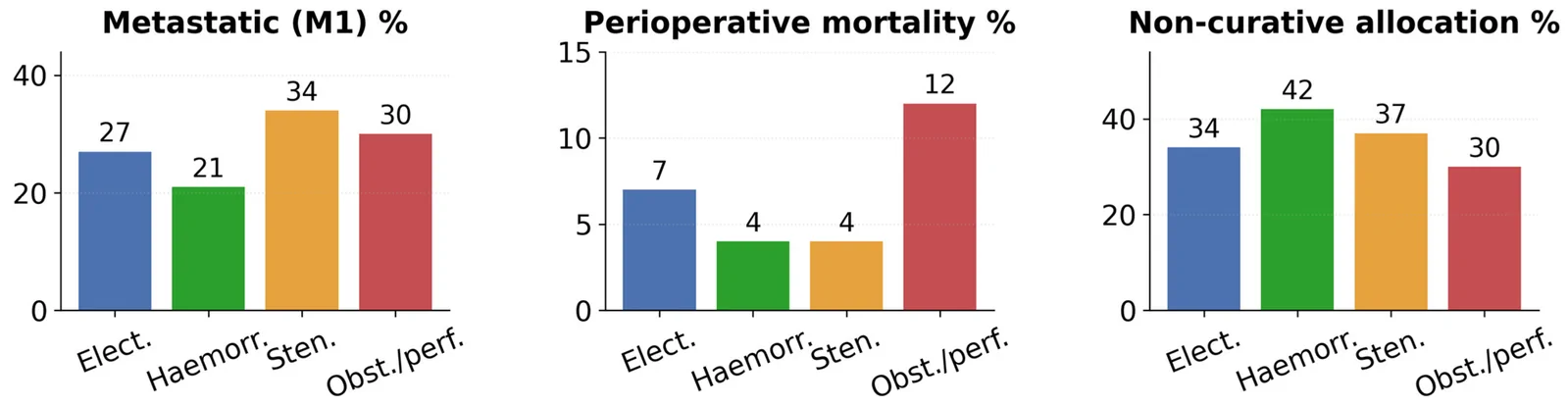
Comparative clinical and treatment profile across emergency diagnostic phenotypes: metastatic disease, perioperative mortality, and non-curative allocation. Elect., elective; Haemorr., haemorrhagic; Sten., stenotic; Obst./perf., obstructive–perforative; M1, metastatic disease.

The clinically decisive difference was in perioperative mortality among operated patients (*p* = 0.033), which was more than threefold higher in the obstructive–perforative group (11.5%, 21/182) than in the haemorrhagic (3.6%, 4/112) and stenotic (4.0%, 3/75) groups, and higher than in elective patients (6.7%, 28/419). Treatment and perioperative outcomes are shown in Table 2 and Figure 1.

### 3.4. Overall Survival by Phenotype

**Figure 2:**
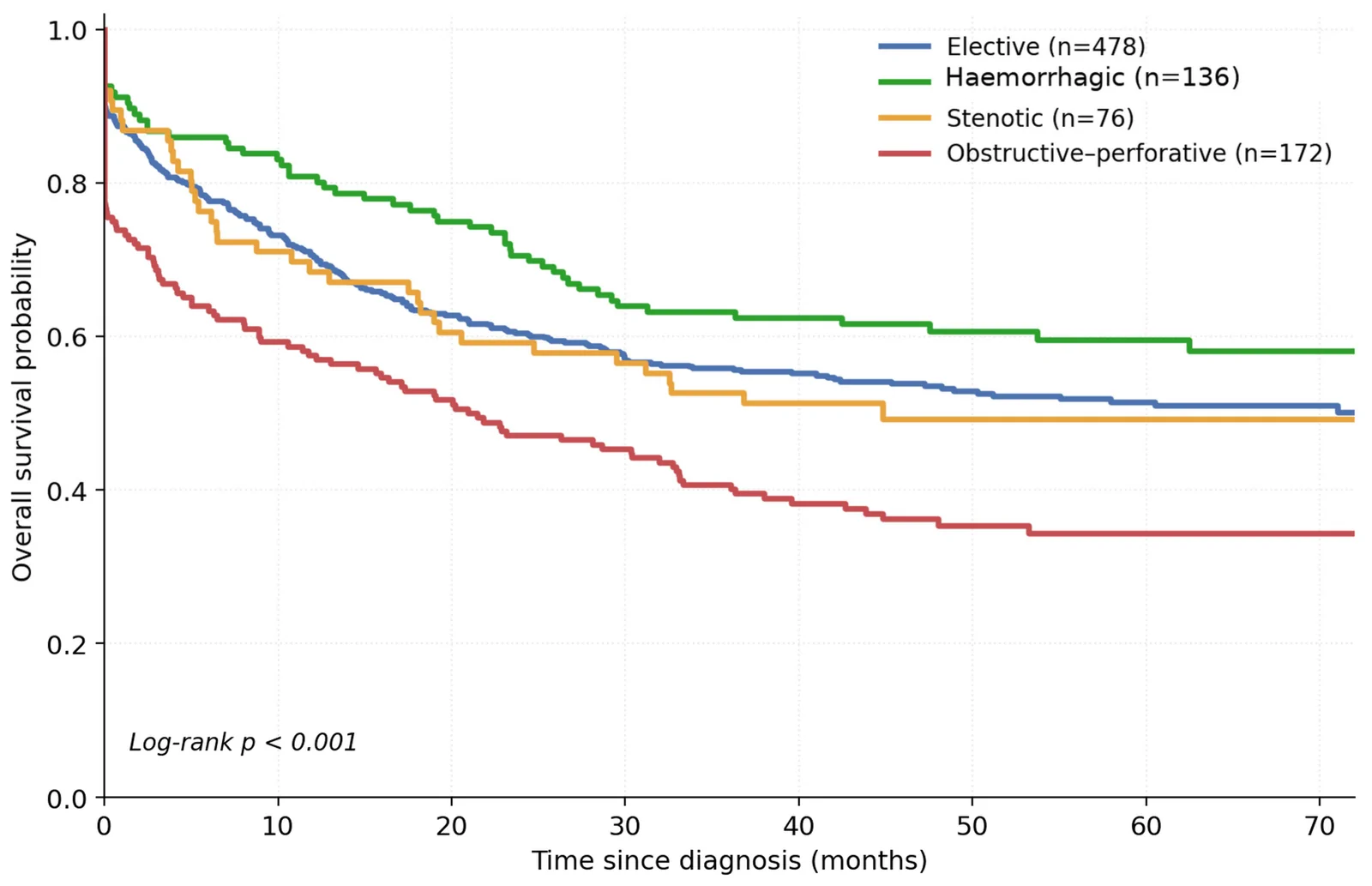
Kaplan–Meier overall survival by emergency diagnostic phenotype.

Overall survival was computed from diagnosis to death or last contact. Among the 862 patients with a documented time-to-event (433 deaths, 429 alive; median follow-up in survivors 1731 days), survival differed markedly across phenotypes (log-rank *p* < 0.001; Figure 2). The obstructive–perforative group had the shortest survival (median 637 days; 12- and 36-month survival rates of 57.6% and 40.7%, respectively). By contrast, the haemorrhagic group showed the most favourable trajectory (median not reached; 12- and 36-month survival 80.9% and 63.2%)—numerically better than elective patients (median not reached; 70.5% and 55.9%). The stenotic group was intermediate (median 1365 days; 68.4% and 52.6%).

Pairwise comparison against elective presentation confirmed the divergence: survival was significantly worse for obstructive–perforative disease (*p* < 0.001), showed a favourable trend for haemorrhagic disease (*p* = 0.075), and did not differ for stenotic disease (*p* = 0.694). Median and time-point survival estimates are given in Table 3.

### 3.5. Independent Predictors of Overall Survival

Formal testing of the proportional hazards assumption showed that metastatic status (*p* < 0.001) and sex (*p* = 0.006) violated it, whereas the three phenotype terms did not (*p* = 0.41–0.48). The primary model was therefore stratified on metastatic status, sex, and age group, after which no covariate violated the assumption. In this primary model (*n* = 859; 431 events), obstructive–perforative presentation remained associated with mortality after adjustment for recorded covariates (HR 1.58, 95% CI 1.25–2.00; *p* < 0.001). Neither of the other emergency phenotypes showed a statistically supported association in the recorded covariate model: the haemorrhagic group showed a non-significant trend towards better survival (HR 0.85, 95% CI 0.63–1.15; *p* = 0.301), and the stenotic group was indistinguishable from elective presentation (HR 1.02, 95% CI 0.71–1.46; *p* = 0.910). Tumour sidedness was not independently prognostic once phenotype and stage were accounted for. The complementary unstratified model gave a closely similar phenotype estimate (HR 1.57, 95% CI 1.25–1.98) and quantified the dominant effect of metastatic disease (HR 3.30, 95% CI 2.71–4.02; *p* < 0.001) and of age (HR 1.03 per year; *p* < 0.001); male sex was of borderline significance (HR 1.21, 95% CI 1.00–1.48; *p* = 0.053). Because metastatic status and sex violated the proportional hazards assumption, these two estimates represent time-averaged effects.

The pandemic-period term was associated with a lower observed hazard (HR 0.73, 95% CI 0.60–0.89; *p* = 0.002). We do not read this as a protective effect; the shorter potential follow-up of patients diagnosed in 2020–2021 mechanically reduces the number of observed deaths, and the phenotype groups were unevenly distributed across the two periods (Table 1). The point is taken up in the Discussion.

**Figure 3:**
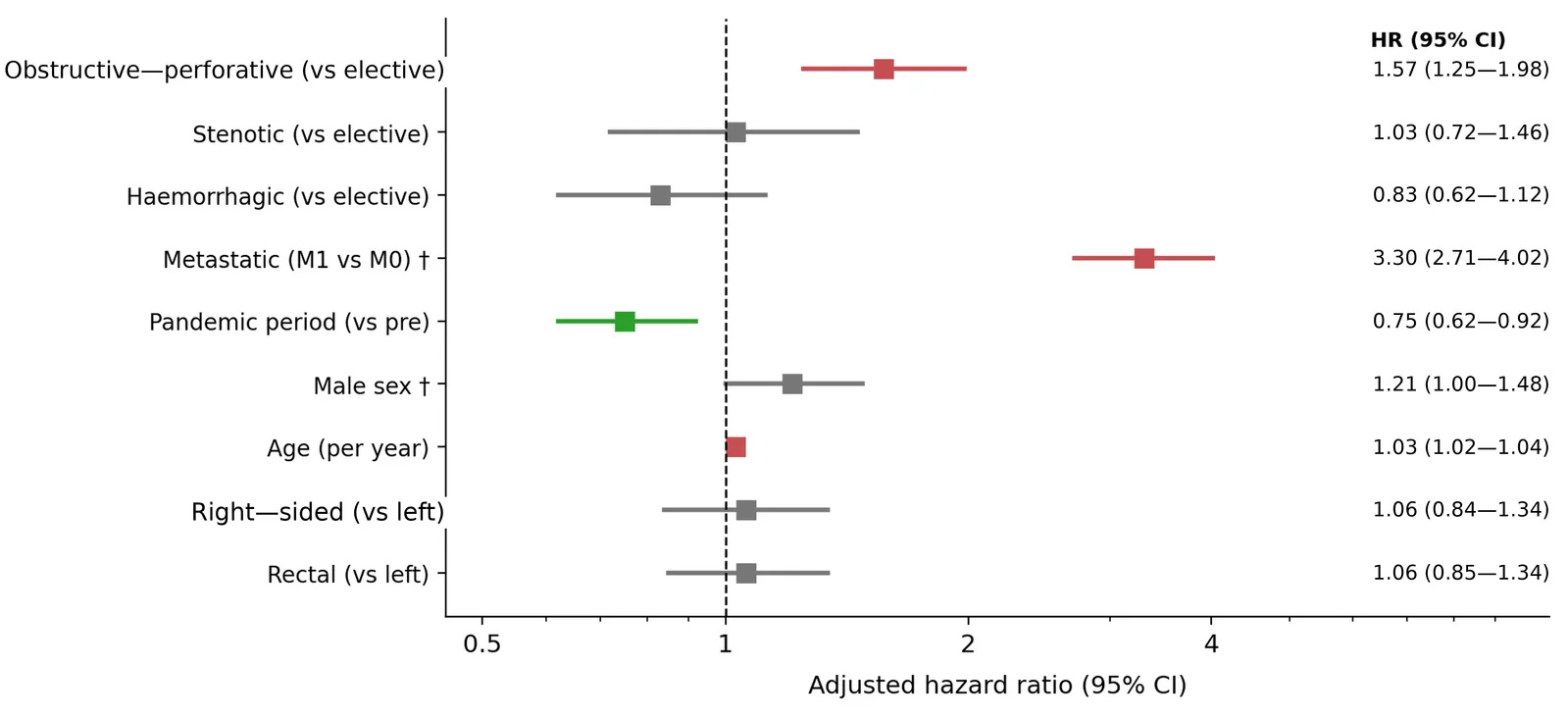
Forest plot of the complementary unstratified Cox model for overall survival. † Proportional hazards assumption violated (Schoenfeld *p* < 0.001 for metastatic status and *p* = 0.006 for sex); the corresponding hazard ratios represent time-averaged effects.

Discrimination of the complementary unstratified model was moderate and stable, with an apparent Harrell’s C-index of 0.699 and a bootstrap optimism-corrected value of 0.691. Both models are summarised in Table 4 and Table 5; the complementary model is displayed in Figure 3.

### 3.6. Predictors of Metastatic Presentation and Non-Curative Allocation

To clarify the mechanism underlying the differences in survival, two complementary logistic models were fitted. In a model for metastatic (M1) disease (*n* = 994), adjusting for age, sex, sidedness, and period, none of the emergency phenotypes was an independent predictor of metastatic presentation (obstructive–perforative OR 1.14, *p* = 0.491; stenotic OR 1.47, *p* = 0.116; haemorrhagic OR 0.74, *p* = 0.155). The excess mortality of the obstructive–perforative phenotype was not explained by recorded metastatic burden, although unmeasured patient-level confounding remains possible. Whether it is fully explained by the acute event and its perioperative consequences is examined in Section 3.7.

In a model for non-curative allocation (*n* = 1047), adjusting for age, sex, metastatic status, and period, metastatic disease was the dominant driver (OR 3.71, 95% CI 2.78–4.95, *p* < 0.001). Independent of metastatic status, the haemorrhagic phenotype was associated with higher odds of non-curative allocation (OR 1.57, 95% CI 1.09–2.26, *p* = 0.016)—consistent with the predominance of rectal cancers managed along nonoperative pathways—whereas the obstructive–perforative phenotype was not (OR 0.79, *p* = 0.217), reflecting near-universal, albeit frequently urgent, operative management. This association was not apparent in the unadjusted comparison (*p* = 0.109; Table 2), and emerges only once metastatic status is held constant.

### 3.7. Sensitivity and Robustness Analyses

**Figure 4:**
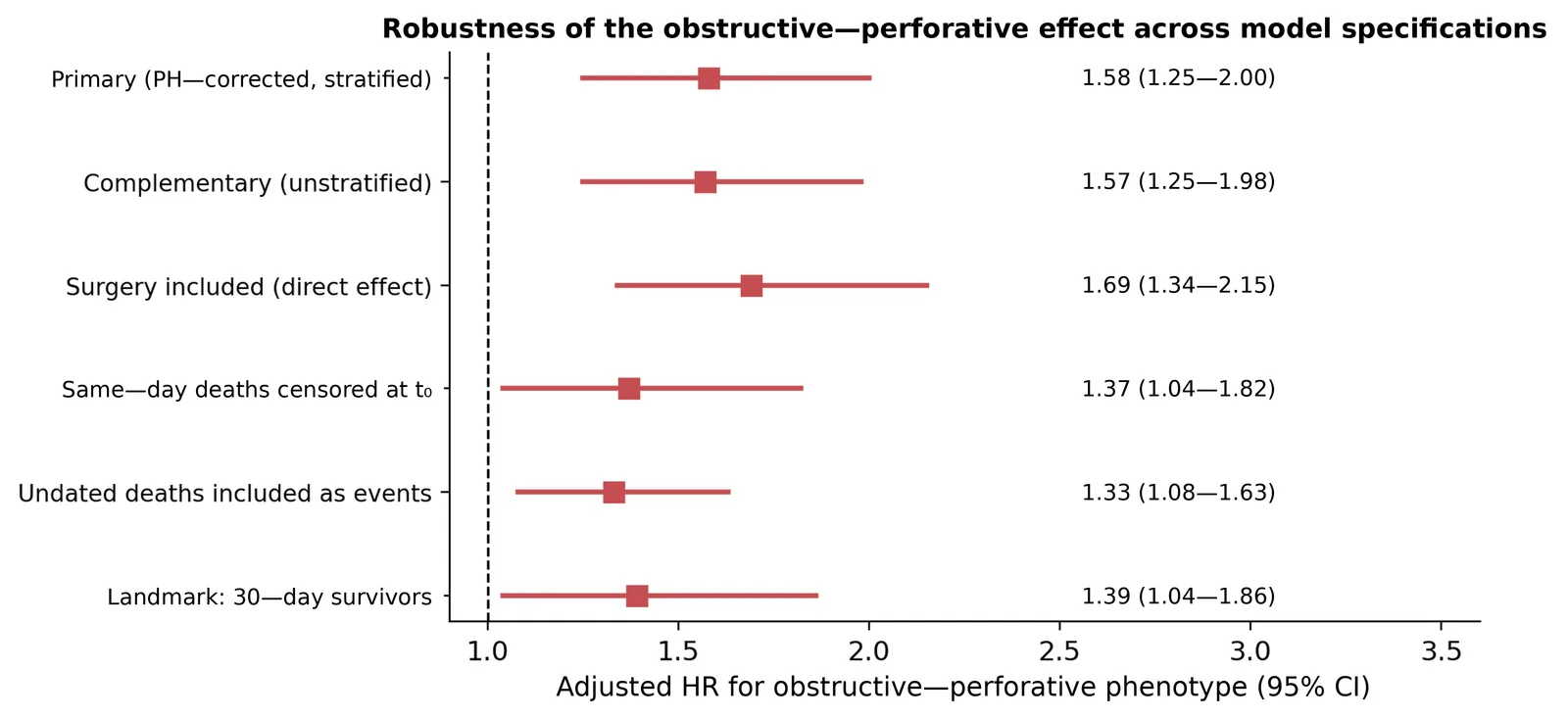
Robustness of the obstructive–perforative effect across model specifications.

Because several analytical decisions could plausibly have shaped the principal finding, each was tested directly. The results are summarised in Table 5 and Figure 4.

Adjustment for surgery. Adding surgery to the primary model—thereby estimating the direct rather than the total effect of phenotype—did not weaken the association; it strengthened it (HR 1.69, 95% CI 1.34–2.15; *p* < 0.001), as expected when a mediator that partly transmits harm is held constant.

Treatment of same-day deaths. Applying a conservative time-zero censoring rule, whereby deaths recorded on the day of diagnosis are censored rather than counted as events, removed 103 events from the analysis. The obstructive–perforative signal persisted (HR 1.37, 95% CI 1.04–1.82; *p* = 0.027).

Patients with undated deaths. A total of 189 patients were known to have died but had no recorded date of death and were therefore absent from the primary time-to-event analysis—almost three in ten of all deaths, and thus a matter of substance rather than bookkeeping. These patients were not concentrated in the high-risk group, which provides partial reassurance but does not establish that inclusion in the survival cohort was non-informative. Undated deaths accounted for 19.3% of elective, 20.0% of haemorrhagic, 20.0% of stenotic, and 11.3% of obstructive–perforative patients (χ^2^ *p* = 0.066); if anything, their exclusion removed proportionally fewer patients from the group in which mortality was highest, a pattern compatible with attenuation of the phenotype estimate; however, the direction and magnitude of selection bias cannot be established without the true event times. When these deaths were treated as events occurring at last documented contact and the analysis repeated in the 1047 patients with complete covariate data, the association was attenuated but preserved (HR 1.33, 95% CI 1.08–1.63; *p* = 0.007).

This analysis should be read as a deliberately conservative stress test rather than as recovery or imputation of the missing event times. It cannot correct the Kaplan–Meier curves or guarantee unbiased absolute survival estimates.

Landmark analysis beyond the perioperative window. To determine whether the survival penalty of obstruction and perforation is exhausted by early operative mortality, the analysis was repeated among the 733 patients who survived the first 30 days after diagnosis, with the time axis reset accordingly, following established landmark methodology [21]. The excess risk was reduced but did not disappear (HR 1.39, 95% CI 1.04–1.86; *p* = 0.028; log-rank vs. elective *p* = 0.023). Median survival among 30-day survivors was 1305 days in the obstructive–perforative group and was not reached in the remaining three groups. Perioperative mortality therefore accounts for part—but demonstrably not all—of the disadvantage borne by these patients.

Across all specifications, the obstructive–perforative estimate remained above 1 and statistically supported, with hazard ratios ranging from 1.33 to 1.69, whereas neither the stenotic nor the haemorrhagic phenotype attained independent significance in any model.

## 4. Discussion

The central message of this study is deceptively simple: in colorectal cancer, an “emergency” at diagnosis is not one thing. When the four classic acute presentations—obstruction, perforation, severe stenosis, and lower gastrointestinal bleeding—are pooled into a single category, as much of the literature and our own initial hypothesis assumed, they cancel one another out and the prognostic signal all but disappears. Once they are separated, a clear and clinically intuitive gradient emerges. Obstruction and perforation behave as a genuinely high-risk, mechanically catastrophic phenotype; haemorrhagic presentation behaves favourably and looks, if anything, better than elective referral; and severe stenosis sits in between. We believe this heterogeneity, rather than the blunt dichotomy of “emergency vs. elective”, is the more faithful description of how these patients actually present and fare.

### 4.1. The Obstructive–Perforative Phenotype

Our finding that obstruction and perforation carry the heaviest prognostic burden is consistent with three decades of the surgical literature. Emergency presentation of colorectal cancer has long been associated with poorer five-year survival [3]; obstruction and perforation are recognised as adverse prognostic features in their own right, independent of stage [5,6]. Contemporary guideline frameworks likewise classify obstruction and perforation as high-risk features in colon cancer, alongside T4 disease, inadequate nodal sampling, and lymphovascular invasion [22,23,24]. Direct comparisons of perforated vs. obstructed colon cancer have generally reported broadly similar overall survival but higher postoperative morbidity and recurrence with perforation [9,10,25]. What our data add is granularity: within a single consecutive cohort, the obstructive–perforative group had the shortest median survival (637 days), the lowest three-year survival (40.7%), and—most tellingly—a perioperative mortality of 11.5%, more than three times that of the haemorrhagic and stenotic groups. The excess risk persisted after adjustment for recorded covariates and after correction for non-proportional hazards (HR 1.58, 95% CI 1.25–2.00), whereas neither of the other emergency phenotypes was independently associated with mortality.

This should not be interpreted as a fully adjusted or causal effect: comorbidity, frailty, performance status, and preoperative physiological severity were not uniformly available and may account for part of the association.

The size and consistency of this signal matter clinically. Population-based work has shown that obstruction, perforation, and emergency admission are not randomly distributed but cluster in patients with less favourable tumour biology and constrained diagnostic pathways [4,26,26,27,28,29]; large multicentre series of malignant large-bowel obstruction have similarly reported substantial operative mortality and poorer long-term outcomes [30,31,32,33]. Our cohort reproduces that picture in a real-world Eastern European setting and suggests that the observed risk is concentrated: it lies in the acute mechanical event, in the surgery it forces, and—as Section 4.3 sets out—in something that outlasts both.

### 4.2. The Haemorrhagic Phenotype

The most counterintuitive—and, we would argue, most interesting—finding is that lower gastrointestinal bleeding did not behave as a high-risk emergency at all. Patients in the haemorrhagic group had the lowest metastatic burden (21.2%), the lowest perioperative mortality (3.6%), and a survival trajectory numerically superior to that of elective patients. One plausible, but unconfirmed, explanation is by the biology of the symptom and its anatomy: over half of haemorrhagic presentations were rectal, and rectal bleeding is a well-recognised early alarm symptom that tends to bring patients to attention sooner and is associated with shorter diagnostic intervals and earlier-stage disease [8,34,35]. In other words, visible bleeding is often a diagnostic gift rather than a marker of catastrophe—the tumour announces itself while it is still, comparatively, curable.

This interpretation also resolves an apparent paradox in our treatment data. The haemorrhagic group had the highest rate of non-curative allocation (41.8%), and this association survived adjustment for metastatic status (OR 1.57, 95% CI 1.09–2.26). Rather than reflecting worse disease, this pattern reflects the predominance of rectal cancers managed along nonoperative or neoadjuvant pathways, in which “no primary surgery” is a deliberate, guideline-concordant strategy rather than a marker of incurability, with watch-and-wait cohorts reporting non-inferior survival compared with resection [36,37,38,39]. The favourable survival of this group, despite lower up-front resection rates, underscores why treatment intent must be interpreted in the light of tumour site and not read mechanically as a severity signal.

### 4.3. Mechanisms: Stage, Surgery, and Residual Risk

A natural objection is that emergency phenotypes might be nothing more than proxies for advanced disease. Recorded metastatic status does not support that as a complete explanation. The proportion of metastatic disease did not differ significantly across the four groups, and in multivariable logistic regression, none of the phenotypes independently predicted M1 status [26,27,40,41,42]. Metastatic disease remained, as one would expect, the dominant determinant of both survival and non-curative allocation—but it acted in parallel with the phenotype effect, not as its conduit. Thus, the association was not reducible to recorded metastatic status, but residual confounding by comorbidity, performance status, frailty, and treatment selection remains possible.

The obvious alternative explanation is the operating theatre. Patients presenting with obstruction or perforation are taken to surgery almost without exception, frequently at night, into an unprepared and sometimes contaminated field, and they die perioperatively at more than three times the rate of their haemorrhagic and stenotic counterparts [43,44,45]. It would be tempting to conclude that the entire survival penalty is discharged in those first days. That conclusion, however, would be too neat. When the analysis was restricted to the 733 patients who survived the first thirty days, the excess hazard was reduced yet clearly persisted (HR 1.39, 95% CI 1.04–1.86; *p* = 0.028), and median survival among 30-day survivors was 1305 days in the obstructive–perforative group while remaining unreached in every other group.

Two mechanisms are therefore at work, and they compound one another. The first is acute and immediate: the physiological insult of obstruction or perforation, and the emergency operation it forces. The second is slower and less visible. It is plausible that tumours that obstruct or perforate are biologically or anatomically more advanced in ways that TNM does not capture—greater local invasion and serosal breach, higher-grade histology, tumour cell dissemination at the moment of perforation, compromised nodal clearance under emergency conditions, and the delayed or diminished adjuvant therapy that so often follows a complicated postoperative course, with each additional four weeks of delay before adjuvant chemotherapy being associated with a measurable survival penalty [39]. That the effect survives adjustment for stage and survives the removal of perioperative deaths suggests that the acute event is both a cause of early mortality and a marker of unfavourable disease that conventional staging does not fully record.

This distinction is more than semantic. If the entire penalty were perioperative, the remedy would lie exclusively in surgical and critical care pathways. Because a residual disadvantage persists well beyond the perioperative period, the case for preventing patients from ever reaching this phenotype—through protected endoscopic access and timely referral—becomes correspondingly stronger. Risk is concentrated at the front door, but it is not confined to it.

Treatment-related confounding also deserves caution. Surgery was excluded from the primary Cox model because it may mediate the effect of an acute presentation, and the surgery-adjusted sensitivity model estimates a different, conditional association. Nevertheless, the available treatment variables were coarse and did not capture neoadjuvant regimens, treatment timing, reasons for nonoperative management, postoperative complications, or subsequent systemic therapy. This is particularly relevant to rectal cancer, for which neoadjuvant and organ preservation pathways can alter both the timing and meaning of primary surgery. The present models therefore cannot separate phenotype-related risk from all treatment selection effects.

### 4.4. Sidedness and Diagnostic Period

Primary tumour location is now an established prognostic and predictive factor in colorectal cancer, with left-sided tumours carrying a modest but robust survival advantage over right-sided tumours in large meta-analyses [11,46]. In our cohort, sidedness tracked phenotype in a biologically coherent way—haemorrhagic tumours were rectal-predominant, obstructive–perforative tumours left colonic-predominant—yet sidedness was not independently prognostic once phenotype, stage, and treatment were accounted for. We interpret this not as a contradiction of the sidedness literature but as a reminder that, in an acutely presenting surgical cohort, the mode of presentation captures much of the prognostic information that location would otherwise carry.

The pandemic-period term warrants careful, honest handling. Diagnosis during 2020–2021 was associated with a lower observed hazard (HR 0.73, 95% CI 0.60–0.89) [47,48,49]. We do not interpret this as a protective effect of the pandemic; the far more plausible explanation is the shorter potential follow-up of patients diagnosed later in the study window, which mechanically reduces the number of observed deaths. Approaches such as restricted follow-up and restricted mean survival time can address this artefact, but our cohort was not designed to quantify pandemic-related disruption. This caution is reinforced by a substantial body of evidence that the COVID-19 pandemic disrupted colorectal diagnostic pathways, reduced screening and endoscopy, and increased the proportion of patients presenting acutely and at more advanced stages [50,51]. Our cohort is not designed to quantify that disruption, and we deliberately treat the period variable as an adjustment covariate rather than an exposure of interest.

The concentration of stenotic presentations in 2020–2021 also raises a specific ascertainment concern. Restricted or delayed endoscopy may have allowed lesions to progress before assessment and may have changed which patients underwent endoscopic documentation, increasing the probability that an examined lesion was labelled impassable. Because symptom-to-diagnosis intervals were not uniformly recorded, we could not distinguish biological progression caused by delay from period-related changes in detection or documentation. The 70.5% pandemic-era proportion in the stenotic group should therefore be regarded as potentially affected by classification bias, and no causal pandemic effect is claimed.

### 4.5. Clinical and Diagnostic Implications

For a diagnostics-oriented readership, the practical import is a refinement of risk stratification at the very front door of the cancer pathway. Treating “emergency presentation” as a monolithic red flag over-triages haemorrhagic patients, many of whom have favourable rectal disease, while under-emphasising the specific lethality of the obstructive–perforative subgroup. That subgroup’s hazard is greatest perioperatively—and to that extent modifiable, whether through surgical and critical care pathways or, in selected left-sided obstructions, through self-expandable metal stenting as a bridge to elective resection [16]—but it is not exhausted there, and the residual disadvantage among 30-day survivors argues for sustained oncological vigilance rather than reassurance once the patient leaves theatre. Recognising the phenotype at presentation—something that requires no molecular assay, only careful documentation of the mode of presentation—could help clinicians calibrate perioperative intensity, informed consent discussions, and postoperative surveillance accordingly. It also strengthens the wider public health argument for protecting endoscopic and referral capacity: the more robust the elective pathway, the fewer patients cross the threshold into the high-mortality obstructive–perforative phenotype [14,15].

In practice, the classification could be used as a communication prompt rather than as a stand-alone decision rule. Obstructive–perforative presentation should trigger early multidisciplinary coordination among colorectal surgery, anaesthesia, critical care, radiology, and oncology, with explicit perioperative risk discussion and planning for oncological follow-up after recovery. Severe stenosis warrants prompt assessment for impending obstruction and review of endoscopic, radiological, and surgical options. Haemorrhagic presentation should not automatically be assigned the same perioperative risk as mechanical catastrophe, but it still requires complete staging and rectal cancer pathway assessment where appropriate. These applications remain provisional and should complement, not replace, established staging, physiological assessment, comorbidity evaluation, and multidisciplinary judgement.

### 4.6. Strengths and Limitations

The principal strengths of this study are its consecutive, real-world design; a substantial single-centre cohort with complete documentation of the mode of presentation and treatment allocation; a mechanistically grounded phenotype classification that made the underlying heterogeneity visible rather than averaging it away; and the systematic testing of the analytical decisions on which the principal finding rests.

Several limitations temper interpretation. First, the design is retrospective and single-centre, which limits external generalisability to other health systems and case mixes. Second, emergency phenotypes were abstracted from clinical, endoscopic, and operative records and were not adjudicated against a standardised prospective template; some misclassification, particularly at the boundary between severe stenosis and early obstruction, is possible. Third, overall survival could be placed on the time axis for 862 of 1051 patients; 189 patients known to have died but without a recorded date of death were necessarily excluded from the primary time-to-event analysis. This is close to three in ten of all deaths and constitutes the principal threat to the internal validity of the survival estimates. Two observations temper that concern, though they do not dissolve it. Undated deaths were distributed broadly evenly across the phenotype groups and were, if anything, least frequent in the obstructive–perforative group (11.3% vs. 19.3–20.0% elsewhere; χ^2^ *p* = 0.066), a distribution compatible with attenuation of the phenotype estimate, although the direction and magnitude of selection bias cannot be established; and when they were reinstated as events at last contact, the association was attenuated but retained (HR 1.33, 95% CI 1.08–1.63). Fourth, our decision to retain same-day deaths as genuine perioperative events—appropriate to a surgical emergency cohort—differs from the stricter time-zero censoring rule; under that rule, the effect persisted (HR 1.37, 95% CI 1.04–1.82). A further caveat concerns the diagnostic period. The phenotype groups were unevenly distributed between the pre-pandemic and pandemic intervals, most conspicuously in the stenotic group, of whom 70.5% were diagnosed during 2020–2021 and who consequently had shorter potential follow-up; the possibility of drift in how stenosis was documented over time cannot be excluded, and survival estimates for that group should be read with corresponding caution. Fifth, metastatic status and sex violated the proportional hazards assumption; we addressed this by stratification, and the phenotype terms themselves satisfied the assumption. However, the hazard ratios reported for those two covariates in the unstratified model are time-averaged and should not be overinterpreted. Sixth, cause of death was not available, so only overall survival could be analysed; no cancer-specific survival or competing risks analysis was possible, and perioperative death—a competing event of particular relevance in a surgical cohort—is embedded within the primary endpoint rather than modelled separately. Finally, several clinically important variables, including standardised comorbidity indices, performance status, and the symptom-to-diagnosis interval, were not uniformly available and therefore could not be incorporated into the models, so residual confounding—especially of the perioperative mortality findings—cannot be ruled out. Taken together, these constraints limit causal interpretation and the precision of absolute survival estimates. The observed between-phenotype heterogeneity was consistent across the reported sensitivity analyses, but whether it is independent of unrecorded patient vulnerability remains unresolved.

External validation is therefore a prerequisite to clinical implementation. Independent multicentre cohorts should prospectively standardise phenotype assignment, comorbidity and performance status measurement, symptom-to-diagnosis intervals, treatment pathways, and mortality ascertainment. Such studies should assess discrimination, calibration, and clinical utility across different referral systems and case mixes before the classification is used for routine risk prediction.

## 5. Conclusions

In this real-world cohort of 1051 consecutive patients with colorectal cancer, the mode of emergency presentation at diagnosis was associated with clinically relevant differences in outcome—but only once its internal heterogeneity was made explicit. Treated as a single category, emergency presentation carried little independent prognostic weight; disaggregated into its constituent phenotypes, it revealed a clear gradient. Obstruction and perforation defined a genuinely high-risk, mechanically catastrophic phenotype, characterised by a perioperative mortality of 11.5% and an adjusted mortality hazard of 1.58 in models limited to recorded covariates that held across six model specifications, whereas lower gastrointestinal bleeding behaved as a favourable, rectal-predominant presentation with survival comparable to—or better than—elective referral, and severe stenosis occupied an intermediate position. Importantly, this survival gradient was not explained by differences in metastatic burden, which was similar across phenotypes. Nor was it exhausted by perioperative death: among 30-day survivors, the excess hazard was attenuated but persisted, a pattern consistent with the acute event contributing to early mortality and/or marking unfavourable disease not fully captured by the recorded variables.

These findings argue for a more discriminating reading of the “emergency” label at the front door of the colorectal cancer pathway. Recognising the presenting phenotype—something that requires no molecular assay, only careful documentation of the mode of presentation—may help clinicians calibrate perioperative intensity, informed consent discussions, and postoperative surveillance, while avoiding the over-triage of haemorrhagic patients who often harbour comparatively favourable disease. The hazard borne by the obstructive–perforative subgroup is greatest perioperatively, and to that extent modifiable, but it is not confined there; the residual disadvantage among 30-day survivors argues for sustained oncological vigilance, and reinforces the wider case for protecting endoscopic and referral capacity so that fewer patients cross the threshold into this high-mortality phenotype. Given the retrospective, single-centre design, these results should be regarded as hypothesis-generating and warrant confirmation in larger, ideally multicentre, prospective cohorts; they offer a candidate framework for risk characterisation that requires external multicentre validation before routine diagnostic or surgical application.

## Figures and Tables

**Table 1:** Baseline and clinicopathological characteristics by emergency diagnostic phenotype.

Characteristic	Elective (*n* = 592)	Haemorrhagic (*n* = 170)	Stenotic (*n* = 95)	Obstructive–Perforative (*n* = 194)	*p*-Value
Age, years (mean ± SD)	66.8 ± 10.6	67.1 ± 10.8	67.3 ± 10.2	68.4 ± 10.6	0.364 ^a^
Male sex, %	59.6	64.1	61.1	57.7	0.639 ^b^
Metastatic (M1), %	27.4	21.2	33.7	29.9	0.122 ^b^
Stage IV, %	26.5	21.2	34.7	30.4	0.072 ^b^
Right-sided, %	27.5	18.8	22.1	25.8	<0.001 ^b^
Left-sided, %	32.3	27.1	36.8	52.6	
Rectal, %	38.5	51.8	37.9	21.1	
High-grade (G3–G4), %	22.9	18.2	23.7	27.3	0.355 ^b^
Pandemic-period diagnosis, %	40.0	40.6	70.5	53.1	<0.001 ^b^

^a^ One-way ANOVA. ^b^ Chi-square test. The *p*-value for tumour location refers to the global comparison of the three-category distribution. Metastatic status and stage group were recorded independently and were discordant in nine patients (Section 2.4); therefore, the M-based and stage-based denominators differ slightly.

**Table 2:** Treatment allocation and perioperative outcomes by phenotype.

Outcome	Elective	Haemorrhagic	Stenotic	Obstructive–Perforative	*p*-Value
Any surgery, %	70.6	65.9	78.9	93.8	<0.001 ^a^
Radical (curative) surgery, %	66.0	58.2	63.2	70.1	0.109 ^a^
Non-curative allocation, %	34.0	41.8	36.8	29.9	0.109 ^a^
Perioperative mortality, % (*n*/*N*)	6.7 (28/419)	3.6 (4/112)	4.0 (3/75)	11.5 (21/182)	0.033 ^a^

^a^ Chi-square test. Radical surgery and non-curative allocation are complementary categories and share the same test. Perioperative mortality denominators are operated patients (*N* = 788).

**Table 3:** Overall survival by emergency diagnostic phenotype.

Phenotype	*n* (Events)	Median OS (Days)	12-Month OS, %	36-Month OS, %	Log-Rank vs. Elective
Elective	478 (228)	Not reached	70.5	55.9	reference
Haemorrhagic	136 (55)	Not reached	80.9	63.2	*p* = 0.075
Stenotic	76 (38)	1365	68.4	52.6	*p* = 0.694
Obstructive–perforative	172 (112)	637	57.6	40.7	*p* < 0.001

**Table 4:** Complementary unstratified multivariable Cox proportional hazards model for overall survival (*n* = 859; 431 events).

Variable	HR	95% CI	*p*-Value
Obstructive–perforative (vs. elective)	1.57	1.25–1.98	<0.001
Stenotic (vs. elective)	1.03	0.72–1.46	0.891
Haemorrhagic (vs. elective)	0.83	0.62–1.12	0.228
Metastatic disease (M1 vs. M0) ^a^	3.30	2.71–4.02	<0.001
Age (per year)	1.03	1.02–1.04	<0.001
Male sex ^a^	1.21	1.00–1.48	0.053
Pandemic period (vs. pre-pandemic)	0.75	0.62–0.92	0.005
Right-sided (vs. left)	1.06	0.84–1.34	0.637
Rectal (vs. left)	1.06	0.85–1.34	0.600

Superscript a: proportional hazards assumption violated (Schoenfeld *p* < 0.001 and *p* = 0.006, respectively); hazard ratios represent time-averaged effects. Surgery was excluded from this model as a mediator on the causal pathway between phenotype and death (see Section 2.5); the model including surgery is reported in Table 5. Apparent C-index 0.699; bootstrap optimism-corrected C-index 0.691. Three patients were excluded for missing age.

**Table 5:** Sensitivity and robustness analyses: adjusted hazard ratio for the obstructive–perforative phenotype (reference: elective) across alternative model specifications.

Model Specification	*n* (Events)	HR (95% CI)	*p*-Value	C-Index
Primary model (PH-corrected, stratified)	859 (431)	1.58 (1.25–2.00)	<0.001	—
Complementary model (unstratified)	859 (431)	1.57 (1.25–1.98)	<0.001	0.699
Sensitivity: surgery included (direct effect)	859 (431)	1.69 (1.34–2.15)	<0.001	0.702
Sensitivity: same-day deaths censored at time zero	859 (328)	1.37 (1.04–1.82)	0.027	0.726
Sensitivity: all patients, undated deaths as events	1047 (619)	1.33 (1.08–1.63)	0.007	0.662
Landmark analysis: 30-day survivors	733 (305)	1.39 (1.04–1.86)	0.028	0.733

All models adjusted for age, sex, metastatic status, tumour sidedness, and diagnostic period. In no specification did the stenotic or haemorrhagic phenotype reach independent significance.

## Data Availability

The data presented in this study are available on request from the corresponding author. The data are not publicly available due to institutional privacy restrictions.

## Abbreviations

The following abbreviations are used in this manuscript:

CI	Confidence interval
CRC	Colorectal cancer
HR	Hazard ratio
OS	Overall survival
